# Mixed methods study protocol for combining stakeholder-led rapid evaluation with near real-time continuous registry data to facilitate evaluations of quality of care in intensive care units

**DOI:** 10.12688/wellcomeopenres.18710.3

**Published:** 2023-11-01

**Authors:** The Collaboration for Research, Implementation and Training in Critical Care in Asia and Africa (CCAA), Aasiyah Rashan, Abi Beane, Aniruddha Ghose, Arjen M Dondorp, Arthur Kwizera, Bharath Kumar Tirupakuzhi Vijayaraghavan, Bruce Biccard, Cassia Righy, C. Louise Thwaites, Christopher Pell, Cornelius Sendagire, David Thomson, Dilanthi Gamage Done, Diptesh Aryal, Duncan Wagstaff, Farah Nadia, Giovanni Putoto, Hem Panaru, Ishara Udayanga, John Amuasi, Jorge Salluh, Krishna Gokhale, Krishnarajah Nirantharakumar, Luigi Pisani, Madiha Hashmi, Marcus Schultz, Maryam Shamal Ghalib, Mavuto Mukaka, Mohammed Basri Mat-Nor, Moses Siaw-frimpong, Rajendra Surenthirakumaran, Rashan Haniffa, Ronnie P Kaddu, Snehal Pinto Pereira, Srinivas Murthy, Steve Harris, Suneetha Ramani Moonesinghe, Sutharshan Vengadasalam, Swagata Tripathy, Tiffany E Gooden, Timo Tolppa, Vrindha Pari, Wangari Waweru-Siika, Yen Lam Minh

**Affiliations:** 1 Institute of Health Informatics, University College London, London, UK; 2 Centre for Inflammation Research, University of Edinburgh, Edinburgh, UK; 3 Mahidol Oxford Tropical Medicine Research Unit, Bangkok, Thailand; 4 Amsterdam Institute for Global Health and Development, Amsterdam, The Netherlands; 5 Department of Medicine, Chittagong Medical College Hospital, Chattogram, Bangladesh; 6 Nuffield Department of Medicine, University of Oxford, Oxford, UK; 7 Department of Anaesthesia and Intensive Care Medicine, Makerere University, Kampala, Uganda; 8 Department of Critical Care Medicine, Apollo Hospitals Educational and Research Foundation, Chennai, India; 9 Department of Anaesthesia and Perioperative Medicine, University of Cape Town, Cape Town, South Africa; 10 National Institute of Infectious Diseases, Oswaldo Cruz Foundation, Rio de Janeiro, Brazil; 11 Oxford University Clinical Research Unit, University of Oxford, Ho Chi Minh City, Vietnam; 12 Uganda Heart Institute, University of Makerere, Makerere, Uganda; 13 D'Or Institute for Research and Education, Sao Paulo, Brazil; 14 Nat-Intensive Care Surveillance, Mahidol Oxford Tropical Medicine Research Unit, Colombo, Sri Lanka; 15 Institute of Applied Health Research, University of Birmingham, Birmingham, UK; 16 Department of Critical Care, Nepal Intensive Care Research Foundation, Kathmandu, Nepal; 17 Centre for Preoperative Medicine, University College London, London, UK; 18 Department of Intensive Care Anaesthesiology, International Islamic University Malaysia, Kuala Lumpur, Malaysia; 19 Department of Planning and Operational Research, Doctors with Africa CUAMM, Padova, Italy; 20 Department of Global Health, Kwame Nkrumah University of Science and Technology, Kumasi, Ghana; 21 Department of Critical Care Medicine, Ziauddin University, Karachi, Pakistan; 22 Intensive Care Medicine, University of Amsterdam, Amsterdam, The Netherlands; 23 General Surgery, Wazir Akbar Khan Hospital, Kabul, Afghanistan; 24 Department of Anaesthesiology and Intensive care, Komfo Anokye Teaching Hospital, Kumasi, Ghana; 25 Department of Community and Family Medicine, University of Jaffna, Jaffna, Sri Lanka; 26 Department of Anaesthesia, The Aga Khan University, Nairobi, Kenya; 27 Department of Targeted Intervention, University College London, London, UK; 28 Department of Pediatrics, Faculty of Medicine, University of British Columbia, Vancouver, Canada; 29 Department of Critical Care, University College London Hospitals NHS Foundation Trust, London, UK; 30 Teaching Hospital Jaffna, Jaffna, Sri Lanka; 31 AII India Institute of Medical Sciences, New Delhi, India; 32 Chennai Critical Care Consultants Private Limited, Chennai, India

**Keywords:** rapid evaluation, quality of care, intensive care, critical illness, low- and middle-income countries, learning health systems

## Abstract

**Background:**

Improved access to healthcare in low- and middle-income countries (LMICs) has not equated to improved health outcomes. Absence or unsustained quality of care is partly to blame. Improving outcomes in intensive care units (ICUs) requires delivery of complex interventions by multiple specialties working in concert, and the simultaneous prevention of avoidable harms associated with the illness and the treatment interventions. Therefore, successful design and implementation of improvement interventions requires understanding of the behavioural, organisational, and external factors that determine care delivery and the likelihood of achieving sustained improvement. We aim to identify care processes that contribute to suboptimal clinical outcomes in ICUs located in LMICs and to establish barriers and enablers for improving the care processes.

**Methods:**

Using rapid evaluation methods, we will use four data collection methods: 1) registry embedded indicators to assess quality of care processes and their associated outcomes; 2) process mapping to provide a preliminary framework to understand gaps between current and desired care practices; 3) structured observations of processes of interest identified from the process mapping and; 4) focus group discussions with stakeholders to identify barriers and enablers influencing the gap between current and desired care practices. We will also collect self-assessments of readiness for quality improvement. Data collection and analysis will be led by local stakeholders, performed in parallel and through an iterative process across eight countries: Kenya, India, Malaysia, Nepal, Pakistan, South Africa, Uganda and Vietnam.

**Conclusions:**

The results of our study will provide essential information on where and how care processes can be improved to facilitate better quality of care to critically ill patients in LMICs; thus, reduce preventable mortality and morbidity in ICUs. Furthermore, understanding the rapid evaluation methods that will be used for this study will allow other researchers and healthcare professionals to carry out similar research in ICUs and other health services.

## Introduction

An estimated five million deaths per year worldwide could be avoided by improving the quality of the delivery of healthcare ^1^. The Institute of Medicine ^2^ defines quality of healthcare as “the degree to which health care services for individuals and populations increase the likelihood of desired health outcomes and are consistent with current professional knowledge". Quality of care is reflected in structures of healthcare services, patient-level processes undertaken, and the outcomes of healthcare interactions ^3, 4^. Recommendations from the Lancet High Quality Health Systems (HQSS) report called for greater investment in systems and processes that strengthen evaluation and improvement of care in low and lower middle income countries (LMICs), and that these systems should reflect and be sensitive to the diverse needs of communities they serve ^4^.

Critical care encompasses healthcare provided to patients with, or at risk of, immediately life-threatening, potentially reversible conditions, irrespective of age, diagnosis, specific patient group or location ^5^. Improving the safety and effectiveness of care for critically ill patients has the potential to substantially reduce preventable mortality ^1^. Efforts to improve the quality of critical care ^6^ in intensive care units (ICUs) internationally, have increasingly focused on ameliorating risk of complications associated with both the critical illness itself, and the healthcare interventions delivered to treat it. These efforts have included minimising duration of invasive therapies, optimising infection control practices and increasing adherence to antimicrobial stewardship practices. Whilst each of these interventions has an established evidence base of improving outcomes across the heterogeneity of critical illness ^7^, such interventions are often poorly implemented and, or consistently adopted in resource constrained healthcare institutions; notably concentrated in LMICs. In addition, patient-centred care and engagement with families and patients to share experiences of critical care is often overlooked despite being of itself, associated with improved outcomes ^8^.

Healthcare providers, including the National Health Service in the UK, have long relied on models of healthcare evaluation including Donabedian’s which measures care provision through the framework of structures, processes and outcomes ^9^. Classically, critical care services have evaluated and benchmarked care using comparisons of this framework between different hospitals (often annually) and within the same institutions over time (often years) ^10, 11^. However, such methods of evaluation rarely provide the granularity of information or the specific contextual factors needed to determine the reasons for poor care and identify opportunities for improvement. Systematic improvement in quality requires identification of the gaps in care (i.e. the differences between care as intended and care as delivered) and an understanding of the underlying determinants. Furthermore, effectiveness of current care delivery, and the likely success of any quality improvement interventions are dependent on the ability to positively and sustainably influence the behaviour of relevant stakeholders ^12^.

Quality of care and success of interventions to improve care is directly attributable to the behaviour of healthcare providers, organisational culture within hospital settings and external factors affecting healthcare accountability ^3, 12, 13^. In LMICs ^14^, further exploration is needed to understand what factors determine healthcare provider behaviours, organisational cultures and patient and public expectations. Acquiring a better understanding of these determinants is essential to inform the design ^3, 12^ and increase the effectiveness of interventions targeted at improving care both in individual ICUs, and more widely within a healthcare system ^15^.

This project aims to leverage the system infrastructure and community of practice established by The Collaboration for Research, Implementation and Training in Critical Care in Asia and Africa (CCAA) to: evaluate the quality of existing care processes in the ICU; identify individual, team and organisational factors determining current care delivery; and assess the likely influence of these factors on future improvement interventions ^3^. This protocol describes the novel methods proposed to undertake this multi-layered, multi-centre evaluation.

## Protocol

### Study design

**Figure 1.**
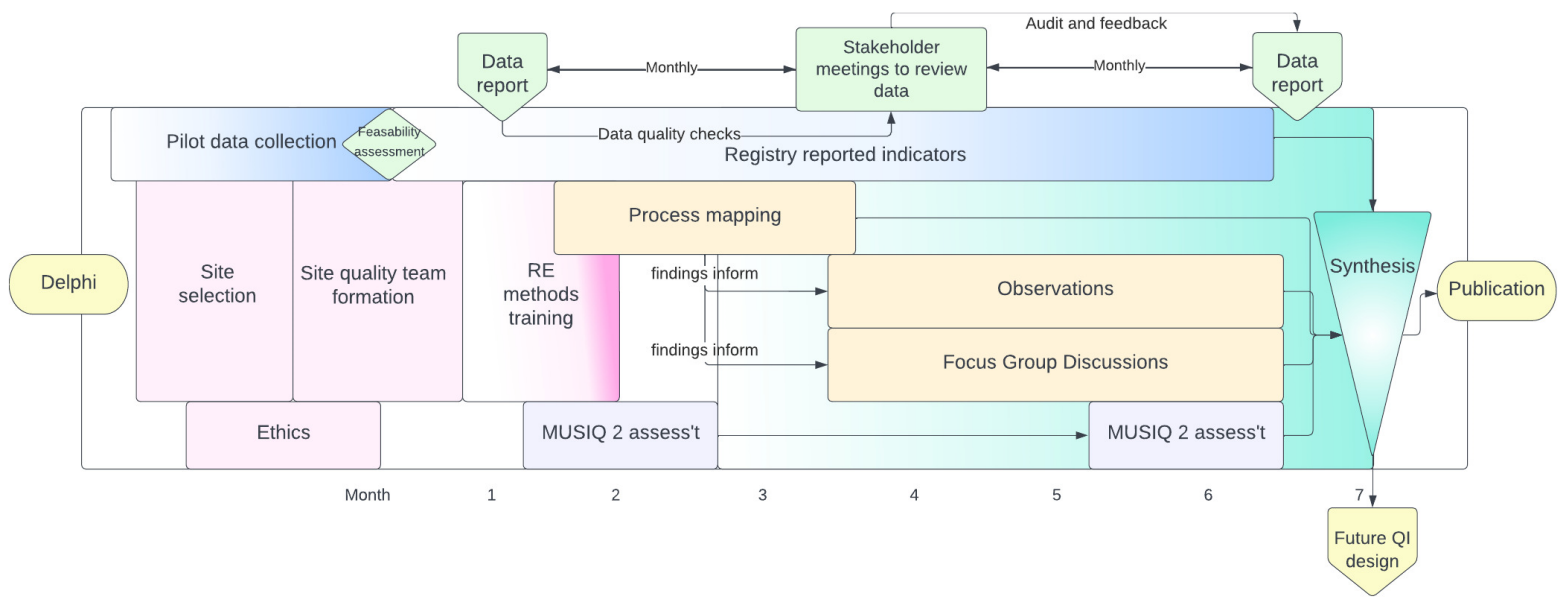
Schema of activities throughout the project.

This is a multi-centre mixed methods rapid evaluation comprising: registry-enabled assessment of care quality using selected process and outcome metrics, stakeholder-led rapid evaluation of the organisational and contextual factors influencing care (process mapping, structured observations and focus group discussions); and an assessment of local quality improvement capabilities ( Figure 1). Together these methods will provide a replicable, comparable and context-specific evaluation of the quality of care processes and their associated outcomes. This evaluation will help stakeholders identify and understand the underlying factors enabling or impeding the delivery and improvement of high-quality care within their departments.

Rapid evaluation (RE) methods ^25, 26^ were chosen for their ability to empower healthcare stakeholders to identify and understand the determinants of existing practice within their own setting ^15^. Process mapping ^27^ conducted by clinical teams, with participation where possible from patients and care receiver representations, will provide a preliminary framework ^28^ to understand gaps between care as intended and care delivered. This information will inform the scope, location and timing of structured observations ^25^ and topics for focus group discussions ^29, 30^.

Structured observations of care will enable the study team to document and describe team and patient-provider interactions relevant to the care process(es) under evaluation. These observations will enable the teams to identify practices of communication, team working and environmental factors (space, location etc) that may affect care delivery. 

Focus group discussions, conducted in parallel to the observations, will be used to further explore and enrich the team’s understanding of the care process(es) of interest from the participants’ view, including their perceptions of barriers to, and enablers of, effective care delivery. 

***Assessment of organisational readiness for care improvement.*** The findings of this multi-centre international evaluation of care will be used to inform future co-design of a toolkit for quality improvement in ICUs. As such, the evaluation will include a replicable assessment of the readiness of participating ICU teams and their institutions to undertake quality improvement activities. The international-validated Model for Understanding Success in Quality (MUSIQ 2) ^31^ calculator designed to identify likelihood of success of future improvement interventions, will be used to gather individual experiences of quality improvement, the site-level data needed to create a shared understanding of local practice and identify local quality improvement capabilities. 

### Setting

**Figure 2.**
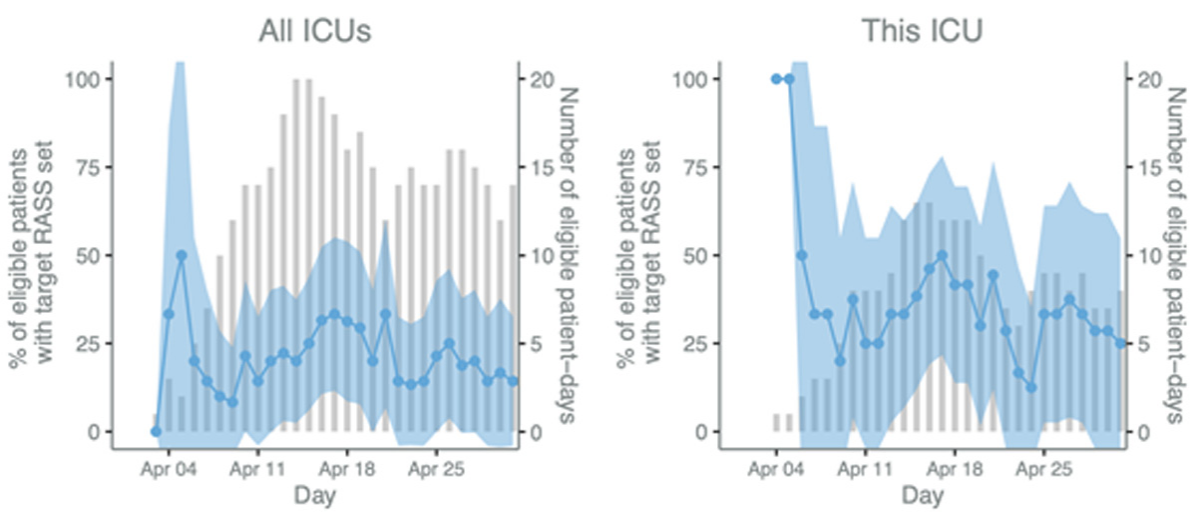
Process evaluation p-charts of Richmond Agitation-Sedation Scale (RASS) rates. The blue line and shaded 95% CI area (left y-axis) display the daily percentage of eligible patients who had a target RASS set and the bars (right y-axis) represent the number of eligible patients. Patients are eligible if they are mechanically ventilated on that day.

The CCAA is supported by a Wellcome Innovations Flagship Programme and UKRI/MRC award, established in 2020, and currently funded until January 2026. The CCAA’s aim is to establish a community of practice equipped with the infrastructure required for a learning health system capable of providing continuous reliable service evaluation, supporting measurable care improvement and facilitating high quality clinical research which translates to practice change. At its core is the ICU digital registry platform which supports a distributed network of clinician-led registries across 17 LMICs, which capture data on case mix, population characteristics, organisational features, care processes and clinical outcomes contemporaneous to the delivery of clinical care for all ICU (and some emergency, perioperative and acute medicine) encounters. The harmonisable data provides near real-time information for service evaluation, clinical research and contextualised sustainable improvements in care delivery ( Figure 2). The registry data set, data collection methods, data quality assurance processes and research impact are described elsewhere ^16, 32– 37^.

CCAA collaborators from ICUs in seven country networks have self-selected to participate in this project. The self-selected countries are Kenya (CCSK), Ghana (KCCR), India (IRIS), Malaysia, Nepal (NICRF), Vietnam and South Africa. Each national network will identify three to six ICUs that have ambition to use their registry for regional and international care quality evaluation, build capacity for health systems research methods and participate in quality improvement interventions. Each participating ICU will also have demonstrated ability to use the ICU registry platform for near real-time daily data collection of ICU encounters inclusive of over 95% of all admissions ^16^. Pluralistic health systems co-exist in each of these countries, whereby private, government and non-governmental organization providers offer critical care services within secondary and tertiary facilities. The CCAA is non-discriminatory in its inclusion of institutions and no representation of diversity of providers is being sought. All country networks and ICUs participate by choice and no financial incentives are used. Factors including care provision and financial models of care may influence organisations’ care quality and ability to engage in quality improvement ^38^. These will be captured and quantified during the project using the mixed methods described above and a future network-wide process evaluation.

### Study team, participants, sample size and recruitment

***Study team.*** Each site will have an ICU quality team consisting of (as a minimum), an ICU nurse, a senior doctor and a data collector. Each ICU team will be supported by their existing national registry coordination team (who include a national lead, coordinator and research assistant). Working alongside these site level and national teams are the already established registry implementation team which includes data scientists, statisticians and clinician researchers with a track record in mixed methods evaluation and service improvement. Together they will provide continued support to the ICU quality team in methods, statistical analysis and overall study conduct. This support is provided where possible remotely, using the Zoom (Zoom Video Communications Inc, San Jose, CA, USA) conferencing application.

***Study participants.*** A wide range of ICU stakeholders will be invited to participate. This will include patients and carers in addition to those responsible for both the strategic service development and delivery of clinical care. We will aim to have balanced representation from each of the stakeholder groups ^39^; however, we recognise the possible limitation of scope from the ICUs in healthcare settings, whereby allied clinical disciplines (e.g. microbiologists, pharmacists, and dieticians) may be limited or poorly represented. Invitation to participate in the project will be sought through the national registry leads and the ICU quality teams.

Stakeholders invited to participate (‘participants’) in the rapid evaluation will have study information made available prior to participation ^17^. Participants will have an opportunity to review the participant information sheet a minimum of 72 hours prior to participation to allow sufficient time for them to consider and seek advice from the ICU or national team or other independent parties. All data collection will be in the language commonly used to deliver healthcare in the setting.

***Sample size and recruitment.*** Sample size will be guided by similar studies and based on achieving sufficient data to explore a range of stakeholder perspectives to understand the factors affecting the specific process of care under evaluation. The combination of purposive sampling of key stakeholders ^25^, including patients, the focused scope of inquiry ^40^ defined by process mapping, and flexible, rapid iterations of data collection and analysis conducted in parallel by teams (a feature of RE method design ^25^) means that the research question may be addressed with estimated 2–3 process maps, 2–3 observations and 2–3 focus groups per ICU ^41^. 

### Data collection

The sequence and timings of the five discrete but complementary sources of data are described in Figure 1.

***Registry enabled continuous evaluation of care***. The components of a learning health system (continuous data for evaluation and new knowledge generation, rapidly integrated into practice) already established by the CCAA will be enhanced by the rapid evaluation, facilitating future practice improvement. The CCAA already has an ICU registry with an established core data set which enables comparable description of case mix, severity of illness and benchmarking of clinical outcomes; the details of the core dataset are described elsewhere. Additionally, the CCAA recently completed a scoping review of ICU quality metrics used internationally and undertook a four round modified Rand Delphi study to identify a set of indicators for evaluation ^21^. Indicators were assessed for their feasibility, reliability, validity to predict outcome and sensitivity to change. They have been classified according to the High Quality Health Systems Framework ^4^: foundations (encompassing human resources, governance structures, accessibility and tools), quality impacts (including clinical and economic outcomes) and care processes (descriptions of care and systems as well as user-experience). These indicators were then prioritised and defined for implementation through the registry. The care processes associated with these priorities are already endorsed by healthcare policy makers in all but the most resource constrained health systems in LMICs ^22^.

The selected metrics are described in Table 1 and reflect previously identified research priorities for improvement: reducing avoidable harms; improving delivery of interventions and processes already proved to improve outcomes and measuring and improving patient-centred outcomes ^21^. Avoidable harms include deep venous thrombosis, stress ulcer, ICU delirium, neuromuscular decline, pressure injury and healthcare-acquired infections. These harms are all associated with prolonged organ support including mechanical ventilation ^8^. The associated daily care processes and interventions proven to ameliorate these harms include optimising sedation and pain management using objective assessment tools (RASS, CPOT); daily assessment of readiness to wake (SAT), assessment of ability to breath spontaneously (SBT) and passive or active mobilization ^23^. Foundations of care, for example nurse:patient ratios, have also been shown to influence these harms. In addition, engagement of family members in daily care in the ICU and their involvement in interventions including respiratory physiotherapy and mobilisation has been demonstrated to improve not only the effectiveness of the interventions, but also to increase both provider and patient compliance, and promote better experience for families and patients ^8^. The feasibility of these measures (three foundation, eight quality impacts and eight care processes) and their definitions ^17^ are currently being assessed ^9, 18^ for feasibility of collection (using the Khan quality framework assessment criteria for conformance and completeness) ^19^ in pilot ICUs in collaborating registries including IRIS (Indian Registry of IntenSive care), NICRF (Nepal Intensive Care Research Foundation), CCSK (Critical Care Society of Kenya) and South Africa.

**Table 1.** Selected measures for registry enabled evaluation.

Foundations	1. Nursing staff to patient ratio 2. Intensivist staffing to bed ratio 3. ICU medical night coverage
Quality impacts	1. Antimicrobial usage, (days of therapy, duration of empirical antimicrobial use) 2. Incidence of ICU-acquired drug resistant organism of interest (DRI) 3. Incidence of HAI (Central Venous Catheter Associated Infection, Catheter Associated Urinary Tract Infections & Infection-related Ventilator-Associated Complication (IVAC)) 4. Incidence of unplanned ICU discharge due to financial constraints 5. Unplanned readmission to ICU 6. Standardised mortality rate (ICU & hospital) 7. Length of stay (ICU & hospital) 8. Quality of life at 30 days post ICU
Care processes	1. Venous thromboembolism prophylaxis 2. Duration of mechanical ventilation 3. RASS score (target and actual) 4. Stress ulcer prophylaxis 5. Spontaneous awakening trial 6. Spontaneous breathing trial 7. Incidence of new pressure sores 8. In-bed mobilisation

[i] ICU: intensive care units; HAI: hospital acquired infection; RASS: Richmond Agitation-Sedation Scale ^20^

Table 1 data are captured each day, extracted from patient charts or directly observed by trained data collectors and entered to the registry platform daily during ICU stay. A comprehensive field specification and data collection guide are made available to all ICU teams through the platform and 24-hour online support is available. Data collectors are trained using already published methods. Weekly follow up meetings with the national registry teams and the site quality teams will provide feedback and troubleshooting for data collection and data quality during weeks 1–4, thereafter monthly meetings using a published quality assurance framework ^16^. Census checks with independent admission data are used to monitor cohort inclusion weekly.

***Rapid evaluation***. Process mapping will be locally-led by the ICU quality teams, with the support of the national registry teams who have experience in rapid evaluation methods, and remote support will be provided by experienced researchers in the implementation team. Process mapping will be conducted in months 2–3 of the evaluation and will inform the priorities for observations and focus group discussions. We will use a general process mapping technique rather than one allied to a specific improvement methodology ^42^. Stakeholders will be asked to map out the discrete activities (tasks) associated with locally-selected care processes from the list in Table 1, identify the actors and equipment involved, the interactions, decision making and who are the decision makers, for care as intended, and for care as it is actually delivered. A mapping guide ^17^ will be used as a template. The mappings will provide a structured, visual representation of each care process which will identify deviations from intended practice and inefficiencies in existing care processes.

Structured observations will be conducted by the ICU quality team, in the clinical area relevant to the processes of interest identified through the process mapping (months 3–6). Data from the observations will be collected using a structured observation guide ^17^ to ensure consistency across study teams and sites. Observations will usually take place within the ICU but may on occasion extend to other locations in local pathways for critically ill patients such as emergency departments and inpatient wards.

Focus group discussions will be conducted by the national registry teams familiar with the context but not directly responsible for care, with support of experienced researchers. Separate focus group discussions will be conducted for healthcare providers and patients/carers so as to limit the impacts of social hierarchies of healthcare within the communities participating ^29, 30^. Focus group discussions will be conducted with between six and eight stakeholders at any one discussion. A template to guide discussions has been designed ^17^ but may be iteratively modified based on the findings of the process mapping and structured observations.

***Assessment of organisational readiness***. The national registry teams will work in partnership with the ICU quality teams to complete an assessment of readiness for quality improvement using the MUSIQ 2 calculator ^31^. ICU quality teams will have an orientation session to the tool led by the national leads, and then complete the MUSIQ 2 calculator online. The MUSIQ 2 will be assessed at the start of the evaluation period (month 1) to help the ICU teams to identify the existing contextual factors which may promote or inhibit the success of the quality evaluation and future improvement initiatives. Scores will be reviewed together with the research team to identify opportunities where the CCAA infrastructure could be leveraged to improve quality improvement capability, for example capacity for quality improvement, data infrastructure, workforce focus and resources for quality improvement. The MUSIQ 2 will be reassessed at month six to describe changes in the micro, environmental and organisational factors as a result of engagement in the evaluation. Scores will be collated and stored on an electronic shared drive for each ICU. Scores will be validated by the registry implementation team for consistency. 

### Data analysis and data management

Data collection and iterative analysis will occur in parallel ^25^. Discovery of information will be a reflexive process in which local knowledge is reconstructed through a cycle of data collection, analysis and planning what to examine next ^25^. Given the evaluations will in part be conducted by the ICU quality teams directly responsible for care, the registry implementation team will facilitate debriefing sessions following each data collection procedure to ensure internal biases (present as a result of priori knowledge of the subject area) are discussed and resolved as data collection/analysis continues and conclusions are drawn ^43^.

Data pertaining to case mix and demographics will be reported using standard descriptive statistics. Diagnosis will be classified from SNOMED CT and mapped to APACHE IV ^44^. Risk adjusted outcomes and predicted mortality will be determined using standardised mortality ratio (SMR). Observed mortality is defined as the percentage of ICU patients who die within hospital (same encounter) as a proportion of all ICU admissions. Observed ICU mortality represents the numerator for risk-adjusted ICU mortality (SMR). The ratio between the observed number of deaths and the predicted number of deaths for the case mix of each ICU, computed by indirect standardisation. Predicted mortality will be determined using APACHE II or e-TropICS (a priori selected by the contributing registry) ^45^. Compliance to process measures will be reported for individual patients based on eligibility each day during the ICU encounter. Composite measures of outcome or event indicators will be calculated as per their published and a priori chosen definitions, using data captured either daily, or by event, as appropriate.

Data arising from the process mapping, observations and focus group discussions will be triangulated ^25^ and analysed using an interpretive analysis approach ^46^. Data will be deconstructed and barriers and facilitators to care quality coded using the Consolidated Framework for Implementation Research (CFIR) framework ^47^. The CFIR was chosen for its ability to facilitate exploration of the individual, and team characteristics, and the in ICU organisational and external factors that promote and inhibit the routine incorporation of interventions into everyday clinical practice ^28, 47, 48^. The findings of each round of analysis will be reconstructed using a Rapid Assessment Process (RAP) sheet ^17^, to repackage the different categories and discover the high level themes that cross cut different care processes within and between individual ICUs. The validity of findings will be discussed and checked with process mapping, observation and focus group participants at every site ^49^.

The Zoom conferencing application will be used to automatically save the audio files (as a MP4 file) from the process mapping exercises to a project-designated, password-protected and automatically backed-up shared drive storage space. The Zoom conferencing application will be used as set out by the University of Oxford ‘Guidelines for using Zoom’ ^50^. The titles of video files will not include a participant’s name, but rather the date of the interview and a site code.

Data management will be overseen by the investigators (DW and AB) and the wider CCAA project team. Data from the process mapping, observations and focus group discussions will be captured digitally and identifiers will be removed. RAP sheets will contain anonymised data only and will also be completed digitally. All data will be retained for five years after the publication of the results. All data will be held on a project-designated, password-protected and automatically backed-up shared drive storage space. All data in the UK will be managed in accordance with EU General Data Protection Regulation (GDPR) ^51^ and if outside the UK, in line with country-specific data protection regulations.

### Ethical and regulatory considerations

The focus of the research is on improvement of healthcare services and is not of a sensitive nature, and thus unlikely to evoke feelings of discomfort or emotional distress for participants. The project will be conducted in accordance with relevant national and international guidance and regulations, including the Global Code of Conduct for Research in Resource-Poor Settings ^52^. To ensure that the project is conducted in an ethical manner, this protocol has been submitted to the Oxford Tropical Research Ethics Committee (OxTREC) ^53^. National registry leads in each collaborating country will be responsible for coordinating with their institutional or institute review boards for relevant approvals. All participants will be given a participant information sheet prior to providing written informed consent.

***Public engagement & involvement.*** National leads were consulted in the design of this project. There is existing literature to support the use of the methods proposed for research inclusive of patients including within the populations considered in this context ^14, 26, 54^.

The registry reports were co-designed and developed with national registry leads and piloted for feedback with the multidisciplinary teams in Kenya, Nepal and India. The research team members are currently being trained in process mapping, observations and focus group discussions by investigators (DW and AB) via video conferencing and using a purpose-build quality improvement resource platform made freely available to all CCAA members ^55^.

Quality improvement initiatives designed to improve care for patients and the public will be explored in subsequent research. The findings of this project will be accessible to patients and the public via the MORU Tropical Health Network website ( www.tropmedres.ac).

### Dissemination

The findings will be used to directly inform the development of a toolbox for implementation of quality improvement interventions in LMICs led by clinician- researchers. The findings will be developed into country-specific manuscripts for publication and also shared across the CCAA collaborating countries. A report will be developed for the funders, Wellcome and UKRI/ MRC. Findings will be published as academic publications as open access and presented at academic conferences.

The country network teams with the support of the lead investigators (DW and AB) will lead writing and reviewing of manuscripts, abstracts and any other publications arising from the overall project. These will be equitably published in academic, peer-reviewed literature as open-access and will offer practical learning for others seeking to utilise similar methods in healthcare institutions worldwide for service improvement. Authorship will be based on the set of criteria outlined by the journal and where possible follow the CredIT Taxonomy ^56^, and will acknowledge that this work is on behalf of the collaborating clinicians, patients and families representing healthcare services within the CCAA. The project results will also be published online ahead of peer review using a free access preprint platform in response to the global academic movement to increase equity and access to healthcare research.

## Study status

The study started in August 2022. Due to staggered start-times between countries, data collection will continue until August 2023. No country has yet completed data collection.

## Discussion

Measurement of key quality indicators, which include patient-centred outcomes, to drive forward improvement is increasingly being promoted as part of benchmarking healthcare quality. Continuous evaluation (driven by data generated each day during routine care delivery) undertaken contemporaneously to stakeholder-led exploration of organisational cultures to inform improvement interventions provides the potential to accelerate service improvement. In this study, we aim to simultaneously lay the foundations for a culture of healthcare improvement and establish capacity for future institution-led research through which healthcare providers, families and patients reflect and appraise current practice, to identify problems and seek possible solutions. As a result, this research has the potential to inform sustainable and effective ICU quality improvement in LMICs for both clinical outcomes and patient-family experiences.

Rapid evaluations (REs) can be characterised as: an intensive, team-based investigation that uses multiple methods of data collection; having an iterative process for collection and analysis; and following the principles of participatory action in order to quickly develop a holistic understanding of a programme from the perspective of key stakeholders, providing a potentially effective methods for institution-led but scalable improvement ^25^. Stakeholder-led rapid research can provide timely insight into specific, complex processes and systems from locally-defined perspectives, which if attempted using more traditional ethnographic methods may take many months before conclusions are made, risking disengagement of stakeholders and delaying service improvement ^57^. RE methods, increasingly used in healthcare ^58^ are amenable to enabling involvement of patient and public stakeholders, representation from whom is often absent in research and which is essential if healthcare providers are to better understand the impact of critical illness on individual families and on wider social and economic population metrics ^4^. Attaining understanding of the context ^13, 26^ in which care is delivered allows tailoring and embedding of any subsequent quality improvement interventions with increased opportunity for their success ^13^.

We anticipate that the combination of traditional benchmarking data from the established near real-time clinical registries together with the qualitative approaches of RE which stem from disciplines including anthropology and business, will enable understanding of existing ICU care processes and how organisational factors and health system structures may influence quality of care both at facility level and in relation to individual patient outcomes. For example, objective replicable daily time series data on the completeness of assessment of sedation use, and readiness to breath spontaneously, in each eligible patient, along with daily changes in ICU case mix, acuity, turnover and staffing numbers, will enable stakeholders to unpack (using iterative cycles of analysis and feedback) how internal and external factors affect aggregate and individual quality of individual care processes and their impact on patient outcomes. The multi-dimensional registry data will be fed back in parallel to ongoing evaluation, thereby providing a reliable and replicable measure of process change over time as future improvement interventions are implemented and evaluated for impact ^59^.

The findings of the project will directly inform subsequent local projects aimed at improving patient care and the development of the CCAA registry to enable effective utilisation of data to drive quality improvement for stakeholders and their ICUs. By understanding organisational readiness for improvement, we will enable stakeholders to plan interventions best suited to their local cultures, needs and resources. Further to this, given this project’s participatory nature, and having been co-designed by clinical stakeholders, novice researchers and clinicians will be exposed to new methods. We anticipate this approach will facilitate the building research and quality improvement capacity in the CCAA which will extend beyond this project.

## Data Availability

No underlying data are associated with this article. Figshare: Supplementary materials for 'Mixed methods study protocol for combining stakeholder-led rapid evaluation with near real-time continuous registry data to facilitate evaluations of quality of care in intensive care units'. https://doi.org/10.6084/m9.figshare.21763325 ^17^ This project contains the following extended data: File 1: Data Completion Guide File 2: Mapping Session Guide File 3: Focus Group Discussion Topic Guides File 4: Invitation Email to CCAA Country Site Leads File 5: Participant Information Sheet: Observations File 6: Participant Information Sheet: Interviews File 7: Observation Template Sheet File 8: Rapid Assessment Process (RAP) Sheet Data are available under the terms of the Creative Commons Attribution 4.0 International license (CC-BY 4.0).
