# Supplementary material for: Mixed methods study protocol for combining stakeholder-led rapid evaluation with near real-time continuous registry data to facilitate evaluations of quality of care in intensive care units
Source: Wellcome Open Res. 2023 Nov 1;8:29. Originally published 2023 Jan 19. [Version 3] doi: 10.12688/wellcomeopenres.18710.3 (PMC10638482; doi:10.12688/wellcomeopenres.18710.3)
Supplement: Peer review history

# Reviewer report: Reviewer response for version 3

**DOI:** 10.21956/wellcomeopenres.22532.r69493

**Published:** 2023-11-10

**Authors:** Heather Schacht Reisinger

**Affiliations:** 1 University of Iowa Carver College of Medicine, Iowa City, IA, USA

Thank you for your thorough response to my review. I'm looking forward to reading more about this study in the future.

Is the study design appropriate for the research question?

Yes

Is the rationale for, and objectives of, the study clearly described?

Yes

Are sufficient details of the methods provided to allow replication by others?

Yes

Are the datasets clearly presented in a useable and accessible format?

Yes

Reviewer Expertise:

Ethnographic and qualitative methods, implementation science, telemedicine. critical care, mental health, US veterans

I confirm that I have read this submission and believe that I have an appropriate level of expertise to confirm that it is of an acceptable scientific standard.

# Reviewer report: Reviewer response for version 2

**DOI:** 10.21956/wellcomeopenres.21150.r66196

**Published:** 2023-09-29

**Authors:** Heather Schacht Reisinger

**Affiliations:** 1 University of Iowa Carver College of Medicine, Iowa City, IA, USA

This manuscript provides a description of an ongoing study on quality improvement in ICUs in LMICs. The study protocol is impressive and could make major contributions on the level of global impact in ICU care in LMICs. The multi-level methods are well-described and there is strong integration of various stakeholders, including patients and caregivers.

 My major recommendation is to move the first four subsections under Protocol (Registry enabled…, The selection of measures…, Rapid evaluation, and Assessment of organizational readiness…) toward the end or integrated into the relevant methods into each section (i.e., general overview immediately followed by details of how that element of the evaluations is going to be conducted). Having the overview sections before the details of the protocol led me to ask a lot of questions about how the elements of each of the sections were being carried out. (I had a lot more critiques as I was reviewing the manuscript that I later deleted because it was there, just not where I was expecting it.)

**Registry and Selection of Measures:**

 The set of agile ICU registry platform is impressive, but a few things are unclear from this section of the evaluation. First, is there a core dataset (enabling evaluation of case mix and risk stratified clinical outcomes) AND a new addition of embedded metric(s) to enable daily data collection? Are only the new embedded metrics found in Table 1 or is that a comprehensive set? Also, I am confused by the “recently added the metric for enabling evaluation of care embedded for daily data collection (Table 1)”? It appears there are multiple metric **s** in Table 1. What does “embedded daily data collection” mean? I think this could be cleared up by integrating the section and the details of the methods.

**Rapid Evaluation:**

 The methods for rapid evaluation are well described. One question I had was which clinical areas will the process maps focus on (e.g., sepsis protocol, vent management, etc.)? Is this pre-determined or will each ICU make their own determination of processes to map?

**Organizational readiness:**

 It would be helpful to know how MUSIQ 2 and organizational readiness for care improvement will be integrated into the other components of the evaluation beyond future planning.

**Discussion:**

 Why was the "learning by doing model" introduced in the Discussion? I’m not sure it added anything to the manuscript to bring it up at the end. If it helped in developing the protocol, it would be helpful to have it at the beginning of the manuscript and more fully described. The discussion focuses a lot on participatory research, patient-centeredness, and stakeholder-led evaluation. While this is part of the work described in the protocol, it was not a key theme in until the Discussion. It may not be needed in the Discussion or it should be highlighted more in the Introduction and Abstract. In contrast to this comment, the Discussion should have more on the impact on global ICU quality improvement that is expected from this protocol and the future impacts. This is how the manuscript was framed in the beginning and it did not return enough to this theme and impact at the end. A minor comment: What was meant by “anthropological methods…may disenfranchise stakeholders from accepting findings, or using them to positive influence service improvement.” This is counter to a lot of anthropological research currently being conducted.

Is the study design appropriate for the research question?

Yes

Is the rationale for, and objectives of, the study clearly described?

Yes

Are sufficient details of the methods provided to allow replication by others?

Yes

Are the datasets clearly presented in a useable and accessible format?

Yes

Reviewer Expertise:

Ethnographic and qualitative methods, implementation science, telemedicine. critical care, mental health, US veterans

I confirm that I have read this submission and believe that I have an appropriate level of expertise to confirm that it is of an acceptable scientific standard, however I have significant reservations, as outlined above.

# Response

**Published:** 2023-10-24

**Authors:** Tiffany Gooden

**Affiliations:** University of Birmingham, UK, UK

Dear Dr Heather Reisinger, Thank you for taking the time to review our manuscript. We hope you find our responses to your comments below and the corresponding edits to the manuscript has elevated our protocol for approval.  

1. **Reorganisation of subsections: **Thank you for this suggestion, we have now moved text around within the protocol methods whereby much of the text that was previously under ‘study design’ is now incorporated into the text under ‘data collection’. We hope this makes the description of the methods easier to follow.
2. **Registry and selection of measures:** Table 1 presents the metrics only for evaluation of care; the registry also comprises a core dataset to evaluate case mix and risk stratification. The core dataset is described in detail within a separate publication. We have amended the text to make this clearer. It now reads as follows:
  “The components of a learning health system (continuous data for evaluation and new knowledge generation, rapidly integrated into practice) already established by the CCAA will be enhanced by the rapid evaluation, facilitating future practice improvement. The CCAA already has an ICU registry with an established core data set which enables comparable description of case mix, severity of illness and benchmarking of clinical outcomes; the details of the core dataset are described elsewhere ^16^. Additionally, the CCAA recently completed a scoping review of ICU quality metrics used internationally and undertook a four round modified Rand Delphi study to identify a set of indicators for evaluation ^21^. Indicators were assessed for their feasibility, reliability, validity to predict outcome and sensitivity to change. They have been classified according to the High Quality Health Systems Framework ^4^: foundations (encompassing human resources, governance structures, accessibility and tools), quality impacts (including clinical and economic outcomes) and care processes (descriptions of care and systems as well as user-experience). These indicators were then prioritised and defined for implementation through the registry. The care processes associated with these priorities are already endorsed by healthcare policy makers in all but the most resource constrained health systems in LMICs ^22^. The selected metrics are listed in Table 1 and reflect previously identified research priorities for improvement: reducing avoidable harms; improving delivery of interventions and processes already proved to improve outcomes and measuring and improving patient-centred outcomes ^21^. Avoidable harms include deep venous thrombosis, stress ulcer, ICU delirium, neuromuscular decline, pressure injury and healthcare-acquired infections. These harms are all associated with prolonged organ support including mechanical ventilation ^8^. The associated daily care processes and interventions proven to ameliorate these harms include optimising sedation and pain management using objective assessment tools (RASS, CPOT); daily assessment of readiness to wake (SAT), assessment of ability to breath spontaneously (SBT) and passive or active mobilization ^23^. Foundations of care, for example nurse:patient ratios, have also been shown to influence these harms. In addition, engagement of family members in daily care in the ICU and their involvement in interventions including respiratory physiotherapy and mobilisation has been demonstrated to improve not only the effectiveness of the interventions, but also to increase both provider and patient compliance, and promote better experience for families and patients ^8^. The feasibility of these measures (three foundation, eight quality impacts and eight care processes) and their definitions ^17^ are currently being assessed ^9, 18^ for feasibility of collection (using the Khan quality framework assessment criteria for conformance and completeness) ^19^ in pilot ICUs in collaborating registries including IRIS (Indian Registry of IntenSive care), NICRF (Nepal Intensive Care Research Foundation), CCSK (Critical Care Society of Kenya) and South Africa.”
3. **Rapid evaluation:** The process mapping will focus on locally-selected care processes from the list in Table 1. We have clarified this now in the manuscript, as below.
  “Stakeholders will be asked to map out the discrete activities (tasks) associated with locally-selected care processes from the list in Table 1, identify the actors and equipment involved, the interactions, decision making and who are the decision makers, for care as intended, and for care as it is actually delivered.”
4. **Organisational readiness: **The MUSIQ 2 calculator will be used to enable stakeholders to reflect upon how their organisational culture may influence future quality improvement efforts. We have amended the discussion to make this clearer, as below.
  “The findings of the project will directly inform subsequent local projects aimed at improving patient care and the development of the CCAA registry to enable effective utilisation of data to drive quality improvement for stakeholders and their ICUs. By understanding organisational readiness for improvement, we will enable stakeholders to plan interventions best suited to their local cultures, needs and resources.  Further to this, given this project’s participatory nature, and having been co-designed by clinical stakeholders, novice researchers and clinicians will be exposed to new methods. We anticipate this approach will facilitate the building research and quality improvement capacity in the CCAA which will extend beyond this project.”

**Discussion:**

**Comment 1:** Why was the "learning by doing model" introduced in the Discussion? I’m not sure it added anything to the manuscript to bring it up at the end. If it helped in developing the protocol, it would be helpful to have it at the beginning of the manuscript and more fully described.

**Response 1:** We have removed the text referring to the learning by doing model from the discussion. It now reads as follows. “In this study, we aim to simultaneously lay the foundations for a culture of healthcare improvement and establish capacity for future institution-led research through which healthcare providers, families and patients reflect and appraise current practice, to identify problems and seek possible solutions.”

**Comment 2:** The discussion focuses a lot on participatory research, patient-centeredness, and stakeholder-led evaluation. While this is part of the work described in the protocol, it was not a key theme in until the Discussion. It may not be needed in the Discussion or it should be highlighted more in the Introduction and Abstract.

**Response 2:** We mention participatory research, patient-centeredness and stakeholder-led evaluation in the discussion because these are core parts of the methodology described for this protocol; as mentioned in the introduction, part of the challenge in measuring and improving quality of care in ICUs is involving key stakeholders (i.e. healthcare professionals, patients and carers). The project we describe will overcome these challenges through participatory research and stakeholder-led evaluations. We allude to patient centeredness in the introduction, but the term was not mentioned; we have now added that into the text. We have also amended the abstract and discussion slightly to make this clearer (see below) Abstract: “Methods: Using rapid evaluation methods, we will use four data collection methods: 1) registry embedded indicators to assess quality of care processes and their associated outcomes; 2) process mapping to provide a preliminary framework to understand gaps between current and desired care practices; 3) structured observations of processes of interest identified from the process mapping and; 4) focus group discussions with stakeholders to identify barriers and enablers influencing the gap between current and desired care practices. We will also collect self-assessments of readiness for quality improvement. Data collection and analysis will be led by local stakeholders, performed in parallel and through an iterative process across eight countries: Kenya, India, Malaysia, Nepal, Pakistan, South Africa, Uganda and Vietnam.” Intro: “In addition, patient-centred care and engagement with families and patients to share experiences of critical care is often overlooked despite being of itself, associated with improved outcomes ^8^.” Discussion: “Measurement of key quality indicators, which include patient-centred outcomes, to drive forward improvement is increasingly being promoted as part of benchmarking healthcare quality.”

**Comment 3:** the Discussion should have more on the impact on global ICU quality improvement that is expected from this protocol and the future impacts. This is how the manuscript was framed in the beginning and it did not return enough to this theme and impact at the end.

**Response 3:** Our focus in the introduction and discussion is around quality improvement and the ways in which quality improvement is measured. In the introduction, we discuss the importance of quality improvement in ICUs but the challenges of achieving this in LMICs and the global issue of not including healthcare professionals and patients in such initiatives. In the discussion, we summarise how and why the methods we outline can overcome these challenges, include healthcare professionals and patients, and inform quality improvement initiatives in ICUs in LMICs. We have however added some text to the discussion on the impact we expect this research to have on ICU quality improvement (see below). “In this study, we aim to simultaneously lay the foundations for a culture of healthcare improvement and establish capacity for future institution-led research through which healthcare providers, families and patients reflect and appraise current practice, to identify problems and seek possible solutions. As a result, this research has the potential to inform sustainable and effective ICU quality improvement in LMICs for both clinical outcomes and patient-family experiences.”

**Comment 4:** What was meant by “anthropological methods…may disenfranchise stakeholders from accepting findings, or using them to positive influence service improvement.” This is counter to a lot of anthropological research currently being conducted.

 ​​​​​​​ **Response 4:** The mention of anthropological methods is simply referring to the length of time it takes to make conclusions from anthropological studies and subsequent improvements to services. We have amended the text to make this clearer (see below). “Stakeholder-led rapid research can provide timely insight into specific, complex processes and systems from locally-defined perspectives, which if attempted using more traditional ethnographic methods may take many months before conclusions are made, risking disengagement of stakeholders and delaying service improvement ^57^.”

# Reviewer report: Reviewer response for version 1

**DOI:** 10.21956/wellcomeopenres.20748.r54370

**Published:** 2023-02-06

**Authors:** Maryati Mohd Yusof

**Affiliations:** 1 Center for Software Technology & Management, Faculty of Information Science & Technology, Universiti Kebangsaan Malaysia, Bangi, Malaysia

The authors have outlined a comprehensive protocol for evaluating ICU quality. The methods are clearly described and well-planned. However, more details can be added:

1. The interview agenda is missing;
2. qualitative data analysis needs further clarification in terms of deductive/inductive approach, thematic analysis, coding, and data representation;
3. process mapping specification: general or specific (e.g. Lean or BPM);
4. study quality measures - e.g. member checking, construct validity.

I wish the authors all the best for a very promising study.

Is the study design appropriate for the research question?

Yes

Is the rationale for, and objectives of, the study clearly described?

Yes

Are sufficient details of the methods provided to allow replication by others?

Yes

Are the datasets clearly presented in a useable and accessible format?

Yes

Reviewer Expertise:

Health information systems evaluation, qualitative methods, quality improvement, Lean healthcare

I confirm that I have read this submission and believe that I have an appropriate level of expertise to confirm that it is of an acceptable scientific standard.

# Response

**Published:** 2023-02-15

**Authors:** Tiffany Gooden

**Affiliations:** University of Birmingham, UK, UK

Dear Dr Maryati Yusof, Many thanks for reviewing our manuscript and proving such a positive assessment. Please see below our response to your individual comments.

1. Within our supplemental materials ( https://doi.org/10.6084/m9.figshare.21763325), we have included the topic guide for the focus group discussions (file 3). Here we note that the topics, or agenda will comprise of the following, though these may be iteratively modified based on the findings of the process mapping and structured observations:
  1. For healthcare providers:
    1. Experiences with managing critically ill patients generally
    2. Challenges with specific processes of care for critically ill patients
  2. For patients and family members:
    1. Experiences of ICU generally
    2. Experiences specific to care processes
2. The qualitative data will be analysed using an interpretive analysis approach, a form of thematic analysis, and coded both inductively and deductively using the Consolidated Framework for Implementation Research framework.
3. We have added the following text to the 2nd paragraph of the ‘Data Collection’ section: 'We will use a general process mapping technique rather than one allied to a specific improvement methodology' followed by this reference which describes the methods we plan to use: Vindrola-Padros C, Johnson GA. Rapid techniques in qualitative research: A critical review of the literature. Qualitative Health Research. 2020;30(10):1596-1604. doi:10.1177/1049732320921835. 
4. Please see existing descriptions of triangulation of findings within and between sites in the 3rd paragraph of ‘Data analysis and management’ section. Furthermore, we have added the following text: 'The validity of findings will be discussed and checked with process mapping, observation and focus group participants at every site' followed by this reference: Strauss ME, Smith GT. Construct validity: Advances in theory and methodology. Annual Review of Clinical Psychology. 2009 Apr 27;5:1-25. 
